# Designing, implementing and evaluating an educational program regarding the effects of second-hand smoke in pregnancy on the knowledge, attitude and performance of male smokers

**DOI:** 10.1186/s12978-023-01630-y

**Published:** 2023-06-05

**Authors:** Zohreh Karimiankakolaki, Seyed Saeed Mazloomy Mahmoodabad, Ashraf Kazemi

**Affiliations:** 1 Department of Health, Shahrekord Branch, Islamic Azad University, Shahrekord, Iran; 2 Department of Health Education and Promotion, School of Public Health, Social Determinants of Health Research Center, Shahid Sadoughi University of Medical Sciences, Yazd, Iran; 3 Nursing and Midwifery Care Research Center, Nursing and Midwifery School, Isfahan University of Medical Sciences, Isfahan, Iran

**Keywords:** Male smokers, Pregnancy, Secondhand smoke, Awareness, Attitude, Performance

## Abstract

**Introduction:**

Contact with second-hand cigarette smoke includes inhalation of cigarette smoke caused by the burning of the cigarette itself and inhalation of smoke exhaled by the smoker. Wife's pregnancy can be a motivating factor to change the behavior of men who smoke. Therefore, this study was conducted with the aim of designing, implementing and evaluating an educational program regarding the effects of second-hand smoke in pregnancy on the knowledge, attitude and performance of male smokers.

**Methodology:**

The present study is an interventional type of pre-test and post-test. In this phase, sampling from health centers in Isfahan was done randomly between March and July 2019, the participants were 140 smoking spouses of pregnant women, who visited health centers to receive pregnancy care, and were divided into two intervention groups and control were divided. The data collection tool was a researcher-made questionnaire on men's awareness, attitude and performance regarding second-hand smoke. All data were analyzed with SPSS18 software and Chi-score, Fisher, t-test.

**Results:**

The average age of the participants was 34 years. There was no significant difference between the comparison of demographic variables in the intervention and control groups (p > 0.05). The results of the paired t-test for comparing before and after the training showed that the average score of the emotional dimension of attitude in the two groups of intervention (p < 0.001) and control (p < 0.001), awareness (p < 0.001) and behavior (p < 0.001) was significantly increased in the intervention group after the training, and according to the independent t-test, the average score of the items mentioned after the training in the intervention group was higher than the control group (p < 0.05). Regarding perceived sensitivity (p = 0.066) and perceived severity (p = 0.065), no significant difference was observed.

**Conclusion:**

The awareness, emotional aspect of men's attitude and behavior regarding secondhand smoke increased, but the perceived sensitivity and severity in this regard was not significant despite the increase, so the current training package is effective in training, but considering more training sessions It is necessary with more concrete training with model or training videos in order to improve the sensitivity and perceived intensity of men.

*Trial registration*: Registration of this randomized control trial has been completed with the Iranian Registry of Clinical Trials, IRCT20180722040555N1.

## Introduction

In addition to the high prevalence of smoking as a health problem, the threats caused by cigarette smoke for people who are exposed to it are a double problem that can be pondered. Contact with cigarette smoke includes the inhalation of cigarette smoke caused by the burning of the cigarette itself and the inhalation of smoke exhaled by the smoker [1, 2]. The highest exposure to secondhand smoke was in Eastern Europe, Western Pacific and Southeast Asia with more than 50% of the population exposed [3]. Although the prevalence of smoking for women is low in Iran, the high prevalence of smoking for men exposes women to complications caused by contact with cigarette smoke [4]. In a study in Isfahan, 23.1% of pregnant women were exposed to secondhand cigarette smoke from their husbands at home [5]. Its complications are significant especially in pregnant women, risks including premature birth [6–10], water sac rupture [11], possibility of cesarean birth [7], decreased fetal growth and intrauterine growth delay [6, 10, 12], low fetal weight [8, 9],fetal distress [8, 9], smallness for gestational age [7], sudden infant death syndrome [6, 10]and increased cotinine level in the follicular fluid of exposed women with cigarette smoke [13]. Spouse smoking is a source of exposure to tobacco smoke [14, 15]. The World Health Organization recommends that health care providers should have a minimum recommendation for all pregnant women to avoid exposure to secondhand smoke of any type of tobacco and to encourage family members to quit smoking [16]. Educational interventions regarding smoking have been able to reduce the smoking rate of men during their wife's pregnancy, so the wife's pregnancy can be a motivating factor for changing the behavior of male smokers [17]. Sahebi et al.’s study showed that educating smoking husbands about second-hand smoke during pregnancy is effective in improving their health beliefs and reducing the exposure of pregnant women [18].

Men are on the sidelines of mothers' services and do not have access to information that helps them make informed decisions and protect and improve the health of their spouses, they have a special role in improving the health of mothers, and if they were considered an obstacle in the past, Today, they are part of the solution [19]. Designing intervention programs regarding second-hand smoke plays a role in preventing exposure of pregnant women to cigarette smoke and reducing smoking in men [20]. The study of Mutalib et al., showed that men's smoking behaviors at home were formed due to lack of awareness and understanding of the health risks associated with exposure to SHS and appropriate interventions are necessary [21]. In a research in Karachi, Niser concluded that awareness about the dangers of smoking is low, and only 22% of cigarette smokers are aware of the dangers of cigarette smoke on the people around them [22]. In explaining the factors related to the contact of pregnant women with cigarette smoke, research shows that lack of awareness of the effects of cigarette smoke on family members is one of the important factors [23].

Pregnancy provides the best opportunity for health care providers to help the smoking spouses of pregnant women with the necessary training to understand the complications and threats caused by second-hand smoke and to correct their behavior and by motivating men's participation in care and the reproductive health of their spouses to help reduce smoking around pregnant women, so the present study was conducted with the aim of designing, implementing and evaluating an educational program regarding the effects of second-hand smoke in pregnancy on the knowledge, attitude and performance of male smokers.

## Methods

The current study was an intervention type (pre-test-post-test), the study population was the smoking spouses of pregnant women who referred to health centers in Isfahan city between March and July 2019. The sample size according to a similar study [8] and considering the significance level (α = 0.05) and the power of the test (β = 80%) and the standard deviation of the second-hand smoke rejection behavior score (S = 6) And in order to achieve a significant difference in the average score of behavior in the intervention and control groups, at least 3 points, the number of 63 people and including the drop of 10%, the number of 70 people in each group was considered.

The criteria for entering the intervention include women being at least in the second trimester of pregnancy, men smoking (at least one cigarette per day around a pregnant woman), having at least third middle school education, not participating in other official educational or research programs regarding the protection against secondhand smoke, the desire of pregnant women was to continue receiving prenatal care until delivery through these health centers, and the criteria for leaving the intervention included the absence of the spouse at home for more than a week and termination of pregnancy.

The sampling method was that among the health centers of Isfahan city, 5 centers from the upper city, the lower city and the city center were randomly selected, and the number of 140 male smokers whose pregnant wives were among the clients of the health centers and other conditions of inclusion in the study had, were invited to the study, then they were randomly assigned to intervention and control groups by lottery. After completing the questionnaire and written informed consent in the pre-test phase, the training package was presented to the people of the intervention group, and two months after the intervention, the post-test was conducted. The control group, which initially received no educational intervention, received the educational package at the end of the study due to ethical considerations.

The tool used was a researcher-made questionnaire on the knowledge, attitude and behavior of smoking spouses of pregnant women, which was completed by the participants as a self-report. To determine the validity of the content quantitatively, the questionnaire was given to 10 panelists of experts in health education and health promotion, and reproductive health, and CVR = 0.87 and CVI = 0.88 were obtained, and its Cronbach's alpha reliability for the emotional dimension of attitude was 0.96. Perceived sensitivity was 0.97, perceived severity was 0.96, and behavior was 0.91.

The questionnaire created by the researcher consists of four parts: the first part demographic information: (age of men and women, education of men and women, occupation of men and women, economic status), part two: awareness 10 questions (with options yes, no and I don't know) for example (which exposure to cigarette smoke causes fetal growth, premature birth, reduction of fetal head circumference, etc.), third part: attitude in the emotional dimension 3 questions Perceived sensitivity 10 questions and Perceived severity 10 questions (on the Likert scale, completely disagree, disagree, have no opinion, agree, completely agree) for example, the attitude of the emotional dimension (I don't like smoking next to my wife, when I smoke next to my wife I feel tormented) I feel guilty…), for example, perceived sensitivity (your wife is at risk of premature birth due to exposure to cigarette smoke, your fetus will be underweight due to exposure to cigarette smoke…), for example, perceived severity (Fetal underweight due to the risk of cigarette smoke disrupts its growth, giving birth prematurely due to exposure to cigarette smoke is very dangerous and serious…) and the fourth part: Behavior 8 questions (with a Likert scale, not at all, a little), to some extent, a lot and very much) for example (not smoking around your pregnant wife, smoking in the open space of the balcony or yard away from your wife, etc.).

The educational program regarding exposure to second hand cigarette smoke was provided to the intervention group and the control group received no intervention-based program. Studying the educational package according to the busyness and time limit of the audience, including a 30–60 min educational-explanatory lecture session, educational pamphlet (made by a researcher), animation of the harms of smoking (taken from the website of the Ministry of Health), photo of a fetus (made by a researcher), SMS As a reminder, the training of pregnant women (as training assistants) was to support their husbands.

According to the different structures of the questionnaire for teaching information about pregnancy and childbirth, cigarette smoke and the diagnosis of types of cigarette smoke and its side effects for pregnancy and the fetus, there were protective recommendations against cigarette smoke, which were presented in the form of a speech to increase awareness and attitude.

Showing animation and pictures and presenting an educational pamphlet (with the content of recognizing the effects of second-hand smoke and protective strategies and reducing consumption) were used to create motivation and increase perceived sensitivity and intensity. Simple steps to quit smoking and sharing the experiences of a smoker who has quit before were used to increase performance.

In order to follow up and maintain the training, at the end of the session, a photo of the fetus was given to the participants, to be exposed to them at home, this photo was "a fetus that asked the father not to smoke". Also, men's mobile numbers were received for sending SMS reminders (at the end of every week for two months). Considering that the time interval between pre-test and post-test was two months, a reminder SMS was sent to the participants every weekend.

Pregnant women were educated to protect themselves from cigarette smoke and also support their husbands to quit smoking, and pregnant women participated in the intervention as "educational partners". The educational content is mentioned in the protocol article of this study [20].

The evaluation of the written media (educational pamphlet) was checked with several indicators, the result of the Readability Assessment of Materials (RAM) readability index was 16.6, which was at an acceptable level. The score obtained from the Suitability Assessment Materials (SAM) index was 85%, which was at an excellent level. The score obtained from the Gunning Fog reading index was 9.6, which is equivalent to the level of the third grade of middle school and was in accordance with the entry criteria. The score obtained from the index of determining the educational level of Clouse was 94%, which showed that the text is at an independent level and the learners are able to learn it without the help of the teacher and others.

Data analysis was done using SPSS 18 software, considering the normality of the data, paired t-test, independent t-test, and chi-score were used, and the significance level was considered less than 0.05. This study is the result of the PhD research thesis of health education and health promotion with code of ethics IR.SSU.SPH.REC.1396.133. All the participants were assured that the obtained information would remain confidential, and the objectives of the research were explained to them, and written informed consent was obtained. Registration of this randomized control trial has been completed with the Iranian Registry of Clinical Trials, IRCT20180722040555N1.

## Results

In this study, 140 male smokers with pregnant wives participated and were randomly assigned to two intervention and control groups. The results of comparing the frequency distribution of demographic variables in the two groups before the intervention did not show any significant difference (p > 0.05). The demographic information of the intervention and control groups is listed in Table 1.

**Table 1:** Frequency distribution of demographic and contextual variables of participants in two groups before the intervention

Groups	Intervention		Control		P-value T-Test
	Mean	SD	Mean	SD	
Variables					
Woman's age	29.11	5.08	30.37	5.33	0.156
Man's age	34.54	6.34	34.15	5.74	0.707

	N	%	N	%	χ^2^ p_value_
Variables					
Female education					
High school	17	24.6	8	12.5	0.706
Diploma	43	62.3	42	63.3	
University	9	13.1	16	24.2	
Male education					
High school	15	21.7	15	21.4	0.893
Diploma	46	66.7	45	64.3	
University	8	11.6	10	14.3	
Woman's job					
Housewife	52	74.3	55	78.6	0.345
Employed	18	25.7	15	21.4	
Man's job					
Employee	7	10.6	10	15.2	0.644
Freelance job	44	66.7	41	62.1	
Worker and unemployed	15	22.7	15	22.7	
The economic situation					
Weak	10	15.6	11	16.7	0.745
Medium	47	73.5	45	68.2	
Good	7	10.9	10	15.1	

The results of paired and independent t-test comparing the mean score of knowledge, emotional dimension of attitude, perceived sensitivity, perceived intensity and behavior before and after training are reported in Table 2.

**Table 2:** The mean score of knowledge, emotional dimension of attitude, perceived sensitivity, perceived intensity and behavior before and after training

Group	Intervention		Control		T-test
	Mean	SD	Mean	SD	
Variable					
Knowledge					
Before	3.35	2.23	2.58	2.73	0.065
After	7.73	2.66	2.60	1.69	< 0.001
Paired test	< 0.001		0.769		
Emotional dimension of attitude					
Before	9.64	2.23	12.76	1.97	0.904
After	9.60	1.44	11.70	1.37	< 0.001
Paired test	< 0.001		< 0.001		
Percived sussebtibility					
Before	27.11	5.68	28.55	5.14	0.117
After	28.64	3.02	29.42	3.41	0.174
Paired test	0.066		0.147		
Percived severity					
Before	27.31	6.18	28.21	5.85	0.378
After	28.39	5.84	29.09	3.42	0.343
Paired test	0.065		0.185		
Practice					
Before	19.71	4.00	20.28	4.78	0.445
After	23.78	4.45	21.42	4.66	0.004
Paired test	< 0.001		0.063		

The results of the paired t-test for comparing before and after the training showed that the average score of awareness (p < 0.001) in the intervention group, the average score of the emotional dimension of attitude in the intervention group (p < 0.001) and the control group (p < 0.001) and the average score of behavior (p < 0.001) in the intervention group increased significantly after the training, and according to the independent t-test, the average score of the mentioned items after the training in the intervention group was higher than the control group (p < 0.05). Regarding perceived sensitivity (p = 0.066) and perceived severity (p = 0.065), there was no significant difference between the two groups before and after the intervention (Table 2).

## Discussion

In this study, men's knowledge about second-hand smoke increased after training, and it was more in the intervention group than in the control group. In line with these results, in the study of Nichter et al., the results showed that education about the harms of second-hand cigarette smoke and establishing a ban in this regard in the society is effective in reducing exposure to second-hand cigarette smoke [24]. Nisar et al.'s study in Pakistan showed that only 22% of people were aware of the dangers of second-hand smoke and most people were not aware of its dangers [22]. In Wakefield et al.'s study, the results showed that men are largely unaware of the effects of smoking on the fetus, and the lack of awareness of how cigarette smoke can affect the fetus was an obstacle for their wives to quit smoking during pregnancy [25]. In the study of Cosci et al., the lack of knowledge about the harm of second-hand smoke and its negative effects on the fetus were reported as factors influencing smoking [26]. In Drehmer et al.'s study, the results showed that the education and counseling of male smokers whose children were exposed to cigarette smoke led them to understand the dangers of their child's exposure to cigarette smoke [27]. In the study of Simber et al., the results showed that fathers' awareness of the risks and care during pregnancy is effective in their companionship and participation in maintaining the health of mothers [19]. The study of Mutalib et al., showed that men's smoking behaviors at home were formed due to lack of awareness and understanding of the health risks associated with exposure to SHS [21].

Previous studies have shown that training fathers leads to improving their awareness, attitude and performance in the field of family planning, baby health, baby feeding and communication and support to the wife and researchers suggested culturally appropriate education before birth to fathers together with their wives or in a group of fathers [28, 29].

Similarly, previous studies suggested a positive relationship between SHS knowledge and SHS avoidance [30–32]. On the other hand, a number of studies showed that awareness of secondhand smoke does not lead to its avoidance [33, 34].

Therefore, men's lack of knowledge about the effects of cigarette smoke on pregnancy and the fetus is an important factor in pregnant women's exposure to second-hand smoke, and as expected, providing awareness in this regard through an educational package including information, images and animation and support Wives have been able to increase the awareness of men.

In this study, the average score of the emotional dimension of attitude increased in both the intervention and control groups, and the perceived sensitivity and perceived intensity, although there was no significant difference, were associated with an increase in the score. Regardless of the significance of the results, in line with the present study, the results of Sahebi et al.'s study entitled "Training men about second-hand smoke on the level of exposure of their pregnant women" showed that after the training, the average score of perceived sensitivity, perceived severity, barriers Perceived and perceived benefits have increased and the intervention is effective on people's attitudes [35]. The study of Poorolajal et al. regarding the tobacco control program in Iran showed that most of the participants had a good attitude towards tobacco control strategies and they believed that smoking in closed environments and public places causes the loss of other people's rights and law enforcement, public places, and travel are expected to be associated with reduced exposure to secondhand smoke [36]. In the study of Wakefield et al., it is stated that except for men's lack of knowledge about the effects of their cigarette smoke on the fetus, they believe that their smoking habit is unimportant [25].

In another study, it has been stated that the warning about the dangers of second-hand smoke had the least effect among smokers, and low sensitivity to the consequences and dangers of smoking increases the exposure to second-hand smoke [37]. Also, another study emphasized that men's belief that the fetus is protected in the mother's body and the lack of motivation to quit smoking during the wife's pregnancy due to the perception that the child is not yet real, also, concern about the stress of marital disputes related to smoking cessation is an obstacle to not quitting smoking in men with a pregnant wife [25].

Therefore, personal behavior can be dangerous for others, it affects the conscience, since the emotional dimension of attitude is related to the emotions, feelings and conscience of each person, therefore, education about it can have a significant impact, however In the control group, a noticeable change in the emotional dimension of their attitude was observed due to being influenced by the questionnaire questions.

Maung et al.'s study showed that the level of participants' attitude (87%) about SHS was high. It is also important to conduct behavioral interventions [38]. The study of Khazaee-Pool et al. showed that the educational intervention was able to improve the dimensions of the health belief model, including the perceived sensitivity and severity of smoking in students [39]. Regarding perceived sensitivity and perceived intensity, it can be said that men do not have a tangible idea of the fetus inside the womb and therefore, they cannot understand the environmental effects on it properly. Also, the idea that the fetus is protected in the mother's womb or that it has not yet come to life as a real being prevents a person from believing in quitting smoking during his wife's pregnancy. The use of images and animation created an abstract image for them to a large extent, but it seems that in order to create a more realistic feeling in this regard, it is necessary to use more concrete methods, such as showing a movie or using a fetus model. Also, the lack of effect of training on perceived sensitivity and severity can be due to the small number of training sessions for men, which should be considered in future studies.

In the present study, the average score of men's behavior increased after the training. Stanon et al.'s study pointed out that educational interventions regarding smoking were able to reduce men's smoking during their wife's pregnancy, so wife's pregnancy can be a motivating factor for changing the behavior of male smokers [17]. Zhang et al.'s study showed that people's attitude towards the dangers of cigarette smoke for their child is related to preventive behaviors of their child's exposure to secondhand smoke [40]. Also, another study indicated that the implementation of the no-smoking law at home can be promoted as an important culture and show the value of men in the responsibility of protecting the health of their wives and children [24]. In another study, training men in perinatal care programs provided new opportunities to participate in the health of themselves and their families [41]. Women's pregnancy motivates family members to ensure the health of the baby; this also gives them an opportunity to change behavior with long-term goals, the goal of maintaining a smoke-free environment for the whole family and promoting women's rights [42]. The results of Sun et al.'s study showed that knowledge explained 7.9% of the variance of behavior [43]. On the other hand, a number of studies have shown that behavior does not reflect participants' level of knowledge about SHS [31, 33, 34, 44, 45].

Although the behavior cannot be easily changed and there is no linear relationship between the change of awareness, attitude and performance, but providing simple and practical solutions to protect others from second-hand smoke has been able to make a person do it.

Despite the significant results, this research was associated with limitations, such as self-reporting of the questionnaire, access to male smokers due to busy work, which needs to be considered in the future study.

## Conclusion

In this study, awareness, the emotional dimension of attitude, self-efficacy, and behavior of men regarding second-hand smoke increased, but the perceived sensitivity and severity in this regard, despite the increase, was not significant.

Therefore, it is necessary to consider more training sessions and more concrete training with models or replicas of the fetus or training videos in order to improve the sensitivity and perceived intensity of men so that they feel the reality of the fetus and have a correct understanding of the life of the fetus in the mother's womb.

The participation of men is an important strategy in achieving the development goals of the third millennium, such as empowering women and improving the health of mothers. The World Health Organization considers men's participation in maternal health programs to include things such as: facilitating access and use of perinatal care, increasing awareness in perinatal care and participating in childbirth planning, and conducting needs assessment and identifying appropriate strategies for he considers their conflict necessary. Therefore, the use of this educational package is suggested in intervention studies in the field of second-hand cigarette smoke.

## Data Availability

Not applicable.
